# Exogenous silicon alleviates the adverse effects of cinnamic acid-induced autotoxicity stress on cucumber seedling growth

**DOI:** 10.3389/fpls.2022.968514

**Published:** 2022-08-10

**Authors:** Jian Lyu, Ning Jin, Xin Meng, Li Jin, Shuya Wang, Xuemei Xiao, Zeci Liu, Zhongqi Tang, Jihua Yu

**Affiliations:** ^1^College of Horticulture, Gansu Agricultural University, Lanzhou, China; ^2^State Key Laboratory of Arid Land Crop Science, Gansu Agricultural University, Lanzhou, China

**Keywords:** silicon, cucumber, autotoxicity stress, mineral elements, nitrogen metabolism, root morphology

## Abstract

Autotoxicity is a key factor that leads to obstacles in continuous cropping systems. Although Si is known to improve plant resistance to biotic and abiotic stresses, little is known about its role in regulating leaf water status, mineral nutrients, nitrogen metabolism, and root morphology of cucumber under autotoxicity stress. Here, we used cucumber seeds (*Cucumis sativus* L. cv. “Xinchun No. 4”) to evaluate how exogenous Si (1 mmol L^−1^) affected the leaf water status, mineral nutrient uptake, N metabolism-related enzyme activities, root morphology, and shoot growth of cucumber seedlings under 0.8 mmol L^−1^ CA-induced autotoxicity stress. We found that CA-induced autotoxicity significantly reduced the relative water content and water potential of leaves and increase their cell sap concentration. CA-induced stress also inhibited the absorption of major (N, P, K, Ca, Mg) and trace elements (Fe, Mn, Zn). However, exogenous Si significantly improved the leaf water status (relative water content and water potential) of cucumber leaves under CA-induced stress. Exogenous Si also promoted the absorption of mineral elements by seedlings under CA-induced stress and alleviated the CA-induced inhibition of N metabolism-related enzyme activities (including nitrate reductase, nitrite reductase, glutamine synthetase, glutamate synthase, glutamate dehydrogenase). Moreover, exogenous Si improved N uptake and utilization, promoted root morphogenesis, and increased the growth indexes of cucumber seedlings under CA-induced stress. Our findings have far-reaching implications for overcoming the obstacles to continuous cropping in cucumber cultivation.

## Introduction

Continuous cropping obstacle refers to the phenomenon where the successive plantation of the same crop on the same land leads to a decline in crop yield and quality. Continuous cropping obstacle is becoming increasingly severe in agricultural production worldwide, especially in facility cultivation. In particular, the continuous monoculture of cucumber (*Cucumis sativus* L.)—a popular greenhouse vegetable—has caused increasingly prominent continuous cropping obstacles. This poses a major challenge to the sustainable development of cucumber facility agriculture (Xiao et al., 2019). The major reasons for continuous cropping obstacles are soil acidification, soil nutrient imbalance, autotoxicity, and microbial ecological imbalance (Bai et al., 2020; Zhang et al., 2020a). Of these, autotoxicity is a primary cause of continuous cropping obstacles and inhibits plant growth and development through the release of specific chemical substances into the surrounding environment. This mainly occurs through the volatilization and decomposition of plant leachates from the aerial parts, root exudates, and plant residues (Kato-Noguchi et al., 2018). In cucurbits—including cucumber (Xiao et al., 2020), watermelon (Ding et al., 2021), and melon (Zhang et al., 2021)—autotoxicity is caused by the excessive release of autotoxins secreted by the roots. Of these, cinnamic acid (CA) is the most important, and adversely affects plant growth and development through a range of physiological and biochemical changes (Yu and Matsui, 1994). However, current cultivation practices indicate that traditional methods to overcome continuous cropping obstacles are inefficient. For example, in melon cultivation, it usually takes 3–4 years of crop rotation to suppress continuous cropping obstacles caused by factors such as autotoxicity (Hu et al., 2017). However, this traditional practice of long-term crop rotation is increasingly unworkable under the existing cultivation model. Grafting is another agricultural measure that helps overcome obstacles to continuous cropping, but has been shown to adversely affect crop quality (Kyriacou et al., 2017; Miao et al., 2019). Hence, it is of utmost importance to find a new approach to effectively mitigate obstacles to the continuous cropping of cucumber, thus allowing the sustainable production of facility vegetables.

Si is the second most abundant element in Earth’s crust after O_2_, and is now considered a “quasi-essential” element (Al Murad et al., 2020). Si can be absorbed by plant roots in the form of soluble silicic acid at a pH of ≤9 (Abdelaal et al., 2020). The accumulation of Si in plants varies considerably depending on species and genotype. Most plants are non-silicon accumulators; Gramineae and Cyperaceae plants are usually high silicon accumulators; and Cucurbitaceae, Urticaceae, and Cruciferae plants are intermediate silicon accumulators (Mitani and Ma, 2005; Henriet et al., 2006). There is growing evidence that Si is a beneficial element for plants and that its accumulation positively contributes to plant growth and development under both abiotic and biotic stress conditions (Etesami and Jeong, 2018; Arif et al., 2021). Plants exposed to environmental stress respond by producing reactive oxygen species (ROS) that cause varying degrees of oxidative stress. Therefore, most research has focused on the mitigation of oxidative damage in plants through the Si-mediated modulation of antioxidant defense systems (both enzyme-enabled and non-enzyme-enabled components; Kim et al., 2017; Farouk and Al-Sanoussi, 2019). Si facilitates the alleviation of nutrient imbalance-induced stress, which in turn promotes plant growth, development, and yield (Fatemi et al., 2021). In a review, Souri et al. (2021) reported that Si effectively promoted plant seed germination, improved leaf water status and photosynthesis, and enhanced plant yield and quality. In a study on pepper plants, Jayawardana et al. (2014) showed that Si application improved branch and root morphology and improved pepper fruit quality by increasing mechanical strength, fruit hardness, and cuticle thickness.

Si is an environmentally friendly element due to its non-corrosive and non-polluting nature, and can applied to crops without the risk of harmful residues. Therefore, it is considered a high-quality fertilizer for the development of ecologically green agriculture (Etesami and Jeong, 2018). However, relatively few studies have reported the effects of Si on autotoxicity stress (an abiotic stress) in plants. Autotoxicity has been shown to significantly inhibit seed germination and subsequent seedling growth in melon. In particular, autotoxicity significantly reduced the photosynthetic pigment content, photosynthetic rate, transpiration rate, and water use efficiency of melon seedlings, which inhibited their growth (Zhang et al., 2021). Zhang et al. (2020b) demonstrated that appropriate concentrations of Si effectively alleviated autotoxicity stress in melon seeds, mainly by increasing starch degradation and the activities of enzymes such as amylase, superoxide dismutase, catalase, and peroxidase. In view of this, it is particularly important to explore the effects of Si on autotoxicity stress in cucumber. The effects of Si on ascorbate-glutathione metabolism and photosystem II responses in CA-treated cucumber seedlings have also been reported (Meng et al., 2021). However, no studies have reported the effects of Si on the water status, mineral nutrient uptake, N metabolism, and root morphology of cucumber seedlings under conditions of CA-induced autotoxicity stress. In this study, we evaluated the effects of Si on the above-mentioned indicators in cucumber seedlings under CA-induced autotoxicity stress. The results improve our understanding of Si alleviating physiological responses of cucumber seedlings under CA-induced autotoxicity stress and further reveal the mechanisms by which Si regulates CA resistance in cucumber plants. Our findings contribute to a new theoretical basis for overcoming barriers to continuous cropping in the facility-based cultivation of cucumber.

## Materials and methods

### Cultivation of the plant material

The cucumber seeds (*Cucumis sativus* L. cv. Xinchun No. 4) used in this study were purchased from the Gansu Academy of Agricultural Sciences, China. Neat and plump cucumber seeds were soaked for 8 h, sterilized for 15 min with 0.03% potassium permanganate solution, and placed in a Petri dish covered with two layers of filter paper (Wu et al., 2019). The culture dishes were placed in an artificial climate box (RDN-400E-4; Ningbo Dongnan Instrument Co. Ltd., Zhejiang, China) set to 28°C, and the seeds were primed in the dark. Light culturing was performed when the germination rate exceeded 80%. After culturing for 5–6 days, cucumber seedlings with similar growth vigor were transplanted into a hydroponic box (4 plants/box), fixed with sponges, and further cultivated in an artificial climate box. During the cultivation period, the day and night temperatures were 28°C/18°C, the light intensity was 360 μmol·m^−2^·s^−1^, the photoperiod was 12 h, and the relative humidity was 75%. The nutrient solution used was Yamazaki’s special nutrient formulation [3.5 mmol L^−1^ Ca (NO_3_)_2_·4H_2_O, 6 mmol L^−1^ KNO_3_, 1 mmol L^−1^ NH_4_H_2_PO_4_, 2 mmol L^−1^ MgSO_4_·7H_2_O, 70 μmol L^−1^ C_10_H_12_N_2_O_8_FeNa·3H_2_O, 10 μmol L^−1^ MnSO_4_·4H_2_O, and 50 μmol L^−1^ H_3_BO_3_] and was replaced every 2 days.

### Experimental design

We divided cucumber seedlings with two leaves into four treatment groups as follows: (i) CK: normal nutrient solution; (ii) Si: normal nutrient solution with 1 mmol L^−1^ Si; (iii) CA: normal nutrient solution with 0.8 mmol L^−1^ CA; (V) CA + Si: normal nutrient solution with 0.8 mmol L^−1^ CA and 1 mmol L^−1^ Si. The CA concentration for medium stress (0.8 mmol L^−1^ CA) and the optimal Si concentration (1 mmol L^−1^ Si) for the alleviation CA-induced autotoxicity stress were selected based on our preliminary research (Meng et al., 2021). At 10 days after treatment, the cucumber seedlings were frozen with liquid nitrogen and stored at −80°C until further use. Each treatment was repeated thrice.

### Growth indexes and measurement methods

#### Leaf moisture status

The relative water content (RWC) of leaves was determined according to Abdi et al. (2016) and calculated using the following formula:

RWC (%) = [(FW − DW)/(TW − DW)] × 100

where FW is the fresh weight of the leaves (measured immediately after collection), TW is the leaf weight at full expansion (measured after floating the leaf in distilled water for 4 h at 25°C in the dark), and DW is the dry weight (measured after oven-drying the leaves at 70°C to constant weight). Each treatment included three biological replicates (Each replicate contains three leaves).

Leaf water potential was measured using a pressure chamber (Model 3,005; Soil Moisture Equipment Co. Ltd., Goleta, CA, United States; Bhusal et al., 2019), and the cell sap concentration of leaves was measured with an automatic refractometer (Jinan Haineng Instrument Co., Ltd.).

#### Mineral elements

The shoots and roots of cucumber plants were fixed at 105°C, dried at 80°C to constant weight, ground, sieved with a 0.25-mm sieve, and used for the measurement of mineral elements. Samples used for the measurement of total N, P, and K were digested by the H_2_SO_4_-H_2_O_2_ wet digestion method, and those used to measure total Ca, Mg, Cu, Fe, Mn, and Zn were dry-ashed in a muffle furnace. Total N was measured by the Kjeldahl method using a fully automatic Kjeldahl K1100F apparatus (Jinan Hanon Instruments Company, Jinan, China), and total P was measured using the Mo-Sb colorimetric method. Total K, Ca, Mg, Cu, Fe, Mn, and Zn were measured using a ZEEnit 700P atomic absorption spectrometer (Analytik Jena, Germany).

#### N metabolism-related enzyme activity

The activities of nitrate reductase (NR; EC 1.6.6.1), nitrite reductase (NiR; EC 1.7.1.15), glutamine synthase (GS; EC 6.3.1.2), glutamate synthase (GOGAT; EC 1.4.1.14), and glutamate dehydrogenase (GDH; EC 1.4.1.2) were measured using ELISA kits (Shanghai Yaji Biotechnology Co., Ltd.) according to the manufacturer’s instructions. 1 g of chopped plant tissue was added to liquid nitrogen and ground into powder in a mortar. 9 ml of homogenate (0.01 mol/l PBS, pH: 7.2–7.4) was added, and the supernatant was collected after centrifugation. Then add the reagents in sequence according to the kit instructions. Finally, the absorbance at 450 nm was measured with HBS-1096A (DiTie, Nanjing, China) microplate reader. GDH enzyme activity was measured in mU·L^−1^, and all other enzyme activities were measured in IU·L^−1^.

#### Root morphology and growth indexes

The total root length, total root volume, total root surface area, number of root tips, number of branch roots, and leaf area were measured with a WinRHIZO System (WinRHIZO Pro LA2400; Regent Instruments Inc., Quebec, Canada). Plant height was measured with a ruler from the base of the plant to the stem tip, and the stem diameter was measured using vernier calipers. The shoots and roots of the cucumber plants were washed with ultrapure water, dried, and their fresh weights were recorded using an electronic balance. To measure the dry weight, the shoots and roots were oven-dried at 105°C for 15 min and then dried at 80°C to constant weight (Wu et al., 2019).

### Statistical analysis

All tables and figures were created using Microsoft Excel 2010 (Microsoft Inc., Redmond, WA, United States). The data were analyzed by one-way analysis of variance (ANOVA) and significant differences were detected by Duncan’s multiple range test (*p* < 0.05) using the SPSS software (IBM SPSS Statistics for Windows, version 22.0; IBM Corp., Armonk, NY, United States).

## Results

### Effect of exogenous Si on the leaf water status of cucumber seedlings under CA-induced autotoxicity stress

**Figure 1:**
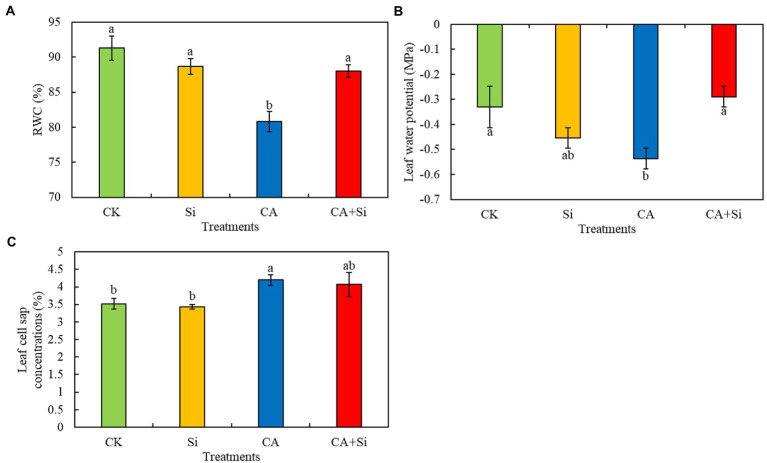
Effect of exogenous Si on the relative water content (RWC; **A**), water potential **(B)**, and cell sap concentration **(C)** of leaves in cucumber seedlings under cinnamic acid-induced autotoxicity stress. Vertical bars represent the mean ± SE from three independent replicates (Each replicate contains three leaves). Different lowercase letters indicate significant differences according to Duncan’s multiple range test (*p* < 0.05).

Treatment with CA significantly reduced the RWC of leaves by 11.48% compared with that of leaves in the CK treatment group (Figure 1A). However, there was no significant difference in RWC between the CA + Si and CK treatment groups. The leaf water potential was significantly lower in CA than in CK. Compared with the CA treatment, the CA + Si treatment alleviated the decline of leaf water potential in cucumber plants under autotoxicity stress. Moreover, the CA + Si treatment maintained the leaf water potential at a level that was not significantly different from that of plants in the CK treatment group (Figure 1B). The concentration of leaf cell sap was 19.32% higher in CA than in CK and 3.10% lower in CA + Si than in CA, although the difference between CA + Si and CA was not statistically significant (Figure 1A). There was no significant difference in RWC, leaf water potential, and leaf cell sap concentration between the Si and CK treatments (Figures 1A–C). These results indicated that CA-induced autotoxicity inhibited the absorption and utilization of water in leaves, reduced the leaf water potential, and increased the concentration of cell sap in leaves, which has adverse effects on their physiological and biochemical reactions. Moreover, the exogenous application of Si was beneficial for maintaining the normal water status of leaves under conditions of CA-induced stress.

### Effect of exogenous Si on the mineral element composition of leaves and roots in cucumber seedlings under CA-induced autotoxicity stress

#### Major elements

**Figure 2:**
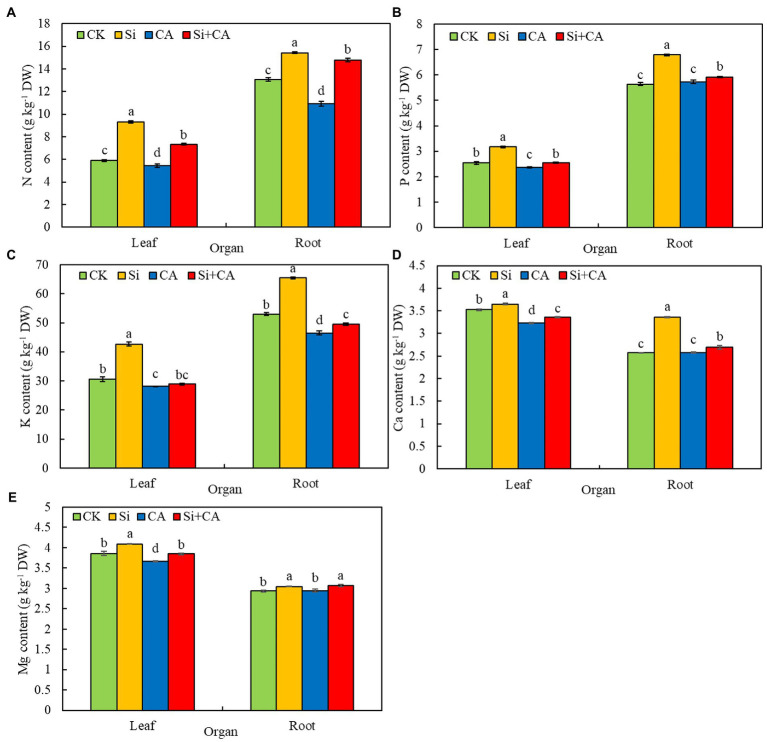
Effects of exogenous Si on the N **(A)**, P **(B)**, K **(C)**, Ca **(D),** and Mg **(E)** contents of leaves and roots in cucumber seedlings under cinnamic acid-induced autotoxicity stress. The values (mean ± SE) represent data from three replicates (*n* = 3; each replicate contains three cucumber seedlings). Different lowercase letters indicate significant differences according to Duncan’s multiple range test (*p* < 0.05).

CA-induced autotoxicity significantly affected the uptake of major elements (N, P, K, Ca, and Mg) by the leaves of cucumber seedlings (Figures 2A–E). Compared with the CK treatment, treatment with CA significantly reduced the N, P, and K contents of cucumber leaves by 7.81, 7.09, and 7.89%, respectively. However, treatment with CA + Si significantly increased the N and P contents of leaves by 34.99% and 7.63%, respectively, compared with those of leaves treated with *CA.* The K content of leaves in the CA + Si group increased by 2.77% compared with that of leaves in CA, but the difference was not significant. The N, P, and K contents of cucumber roots showed similar trends to those of leaves in each treatment group (Figures 2A–C). Compared with the CK treatment, treatment with CA significantly reduced the N and K contents of roots by 16.51% and 12.23%, respectively. Compared with the CA treatment, CA + Si treatment significantly increased the N, P, and K contents of roots by 35.35%, 3.32%, and 6.24%, respectively. In addition, the N, P, and K contents of leaves and roots were significantly higher in the Si treatment group than in CK. Compared with the CK treatment, CA treatment significantly reduced the Ca and Mg contents of cucumber leaves by 8.24% and 4.92%, respectively. However, treatment with CA + Si significantly increased the Ca and Mg contents of leaves by 4.02% and 4.90%, respectively, compared with those of leaves in CA (Figures 2D,E). The Ca and Mg contents of CA-treated cucumber roots were not significantly different from those of leaves in CK (Figures 2D,E). Compared with the CA treatment, CA + Si treatment significantly increased the Ca and Mg contents of cucumber roots by 5.47% and 4.07%, respectively. Moreover, the Ca and Mg contents of leaves and roots were significantly higher in Si than in CK. These results indicated that exogenous Si promoted the absorption of major elements by the leaves and roots of cucumber seedlings, whether in normal conditions or under conditions of CA-induced autotoxicity.

#### Trace elements

**Figure 3:**
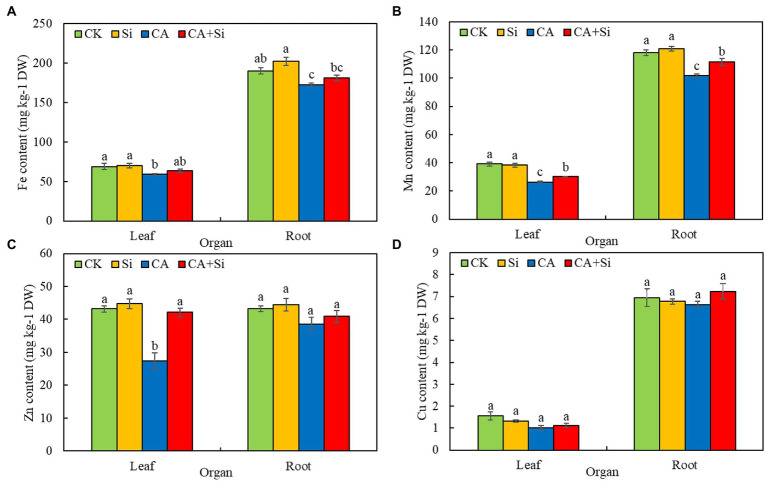
Effect of exogenous Si on the Fe **(A)**, Mn **(B)**, Zn **(C)**, and Cu **(D)** contents of leaves and roots in cucumber seedlings under cinnamic acid-induced autotoxicity stress. The values (mean ± SE) represent data from three replicates (*n* = 3; each replicate contains three cucumber seedlings). Different lowercase letters indicate significant differences according to Duncan’s multiple range test (*p* < 0.05).

The trends of change in trace element composition in each treatment group are shown in Figure 3. CA treatment seriously affected the absorption of trace elements by cucumber leaves. Accordingly, the Fe, Mn, and Zn contents of cucumber leaves were significantly lower in CA than in CK, with a decrease of 14.22%, 32.48%, and 36.52%, respectively (Figures 3A–C). The Fe content of leaves was higher in CA + Si than in CA, but the difference was not significant. The Mn and Zn contents of leaves were significantly higher (by 15.41% and 53.72%, respectively), in CA + Si than in *CA.* The Fe, Mn, and Zn contents of leaves were not significantly different between the Si and CK groups. CA treatment significantly affected the uptake of Fe and Mn by cucumber roots (Figures 3A,B). Compared with the CK treatment, CA treatment significantly reduced the Fe and Mn levels of roots by 9.20% and 5.47%, respectively. The Fe content of roots was higher in CA + Si than in CA, but the difference was not significant. Compared with the CA treatment, CA + Si treatment significantly increased the Mn content roots by 9.65%. There were no significant differences in the Zn content of roots among the four treatments (Figure 3C). The Cu contents of leaves and roots were not significantly different among all four treatments (Figure 3D). These results indicated that exogenous Si alleviated some of the adverse effects of CA-induced autotoxicity stress on the absorption of trace elements by cucumber leaves and roots.

### Effect of exogenous Si on the activities of N metabolism-related enzymes in the leaves and roots of cucumber seedlings under CA-induced autotoxicity stress

**Figure 4:**
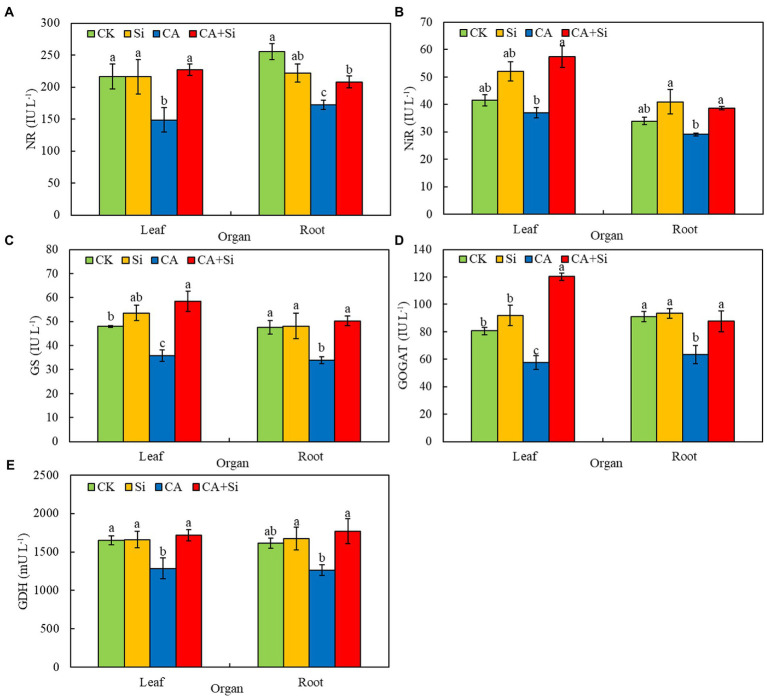
Effect of exogenous Si on the enzyme activities of nitrate reductase (NR; **A**), nitrite reductase (NiR; **B**), glutamine synthase (GS; **C**), glutamate synthase (GOGAT; **D**), and glutamate dehydrogenase (GDH; **E**) in the leaves and roots of cucumber seedlings under cinnamic acid-induced autotoxicity stress. Vertical bars represent the mean ± SE from three independent replicates. Different lowercase letters indicate significant differences according to Duncan’s multiple range test (*p* < 0.05).

The activities of NR, NiR, GS, GOGAT, and GDH in cucumber leaves and roots showed similar trends across the four treatments (Figure 4). Compared with the CK treatment, CA treatment significantly reduced the NR activities of cucumber leaves and roots by 31.43% and 22.47%, respectively. Compared with the CA treatment, CA + Si treatment significantly increased the NR activities of leaves and roots by 52.84% and 20.78%, respectively (Figure 4A). Compared with the CK treatment, CA treatment reduced the NiR activities of cucumber leaves and roots by 10.91% and 14.34%, respectively; however, the differences were not significant. Compared with the CA treatment, CA + Si treatment significantly increased the NiR activities of leaves and roots by 55.27% and 26.11%, respectively (Figure 4B). The NR and NiR activities of leaves and roots were not significantly different between the CK and Si groups (Figures 4A,B).

CA-induced autotoxicity stress significantly inhibited the activities of GS and GOGAT in cucumber leaves and roots (Figures 4C,D). Compared with the CK treatment, CA treatment significantly reduced the GS activities of leaves and roots by 25.28% and 28.71%, respectively (Figure 4C), and the GOGAT activities by 28.62% and 30.27%, respectively (Figure 4D). The exogenous addition of Si alleviated the CA-induced inhibition of GS and GOGAT activities in the leaves and roots of cucumber seedlings. Compared with the CA treatment, CA + Si treatment significantly increased the activities of GS (38.62% and 47.94%) and GOGAT (108.90 and 38.05%) in leaves and roots, respectively. The GS and GOGAT activities of cucumber leaves and roots were not significantly different between the Si and CK groups. Compared with the CK treatment, CA treatment significantly reduced the GDH activities of cucumber leaves and roots by 28.62% and 30.27%, respectively. Compared with the CA treatment, CA + Si treatment significantly increased the GDH activities of leaves and roots by 108.90% and 38.05%, respectively. The GDH activities of cucumber leaves and roots were not significantly different between the Si and CK groups (Figure 4E).

### Effect of exogenous Si on the root morphology and growth indexes of cucumber seedlings under CA-induced autotoxicity stress

#### Root morphology

**Figure 5:**
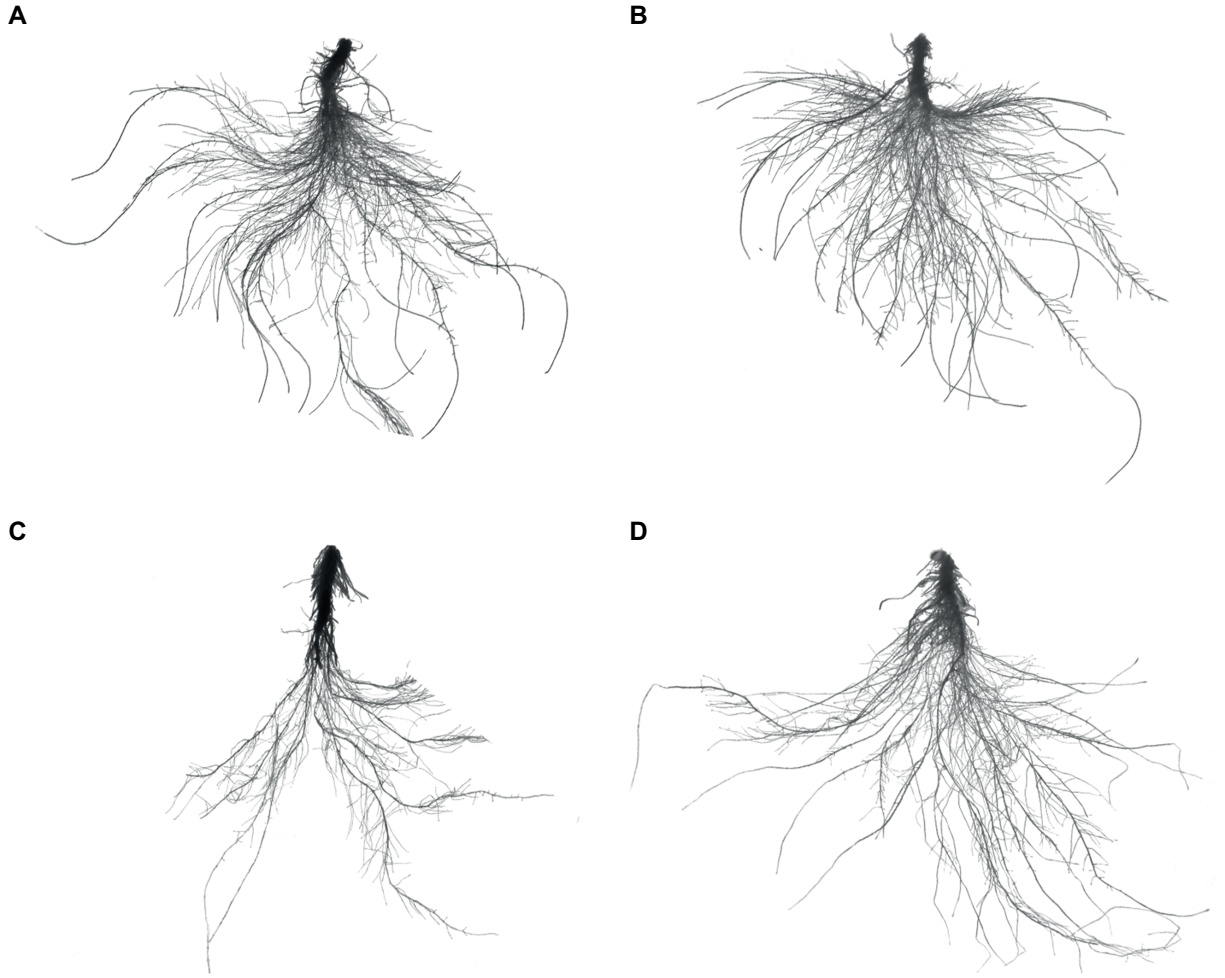
Effect of exogenous Si on the root morphology of cucumber seedlings under cinnamic acid (CA)-induced autotoxicity stress. **(A)** CK: normal nutrient solution; **(B)** 1 mmol L^−1^ Si; **(C)** 0.8 mmol L^−1^ CA; and **(D)** 0.8 mmol L^−1^ CA + 1 mmol L^−1^ Si.

Compared with the CK treatment, CA treatment significantly reduced the total root length, average root diameter, total root volume, total root surface area, and number of branch roots of cucumber seedlings by 33.34%, 25.53%, 50.00%, 45.53%, and 28.39%, respectively (Table 1). In contrast, CA + Si treatment significantly increased the total root length, average root diameter, total root volume, total root surface area, number of root tips, and number of branch roots of seedlings under CA-induced stress by 24.74%, 5.71%, 20.55%, 19.48%, 24.98%, and 15.67%, respectively. There was no significant difference in total root length, total root volume, total root surface area, number of root tips, and number of branch roots between the Si and CK groups. Similarly, CA-induced autotoxicity significantly inhibited root growth (Figure 5C), whereas exogenous addition of an appropriate concentration of Si promoted root morphogenesis (Figure 5D). The root morphology of the Si and CK groups was also similar in appearance (Figures 5A,B).

**Table 1:** Effect of exogenous Si on total root length, average root diameter, total root volume, total root surface area, number of root tip, and number of branch roots in cucumber seedlings under cinnamic acid (CA)-induced autotoxicity stress.

Treatment	Total root length (cm·plant^−1^)	Average root diameter (mm·plant^−1^)	Total root volume (cm^3^·plant^−1^)	Total root surface area (cm²·plant^−1^)	Number of root tips (number·plant^−1^)	Number of branch roots (number·plant^−1^)
CK	994.00 ± 29.02 a	0.47 ± 0.01 a	1.46 ± 0.05 a	140.79 ± 4.46 a	1108.33 ± 55.31 b	5420.92 ± 190.86 a
Si	984.82 ± 68.45 a	0.43 ± 0.01 b	1.37 ± 0.07 a	127.20 ± 8.10 a	1274.83 ± 114.35 ab	5577.58 ± 297.89 a
CA	662.60 ± 38.32 c	0.35 ± 0.00 c	0.73 ± 0.03 c	76.69 ± 4.16 c	1086.33 ± 61.94 b	3881.75 ± 220.86 b
CA + Si	826.50 ± 35.94 b	0.37 ± 0.01 c	0.88 ± 0.05 b	95.24 ± 5.01 b	1357.67 ± 80.55 a	4489.92 ± 270.54 b

Data indicate the mean ± SE (*n* = 3). Different lowercase letters in the same column indicate significant differences according to Duncan’s multiple range test (*p* < 0.05). CK, control.

#### Growth indexes

CA-induced autotoxicity significantly reduced the plant height, stem diameter, and leaf area of cucumber seedlings compared with those of seedings in the CK group (Table 2). Furthermore, CA-induced autotoxicity also significantly reduced the fresh and dry weights of the shoots and roots of cucumber seedlings on the 10th day of treatment. However, compared with CA treatment, CA + Si treatment significantly increased the plant height, stem diameter, and leaf area of cucumber seedlings by 5.87%, 5.29%, and 25.69%, respectively. Compared with CA treatment, CA + Si treatment also significantly increased the fresh and dry weights of cucumber shoots (by 8.00% and 8.81%, respectively) and roots (by 18.21% and 20.04%, respectively). There were no significant differences in plant height, stem diameter, and leaf area between the Si and CK treatment groups. Si treatment significantly increased the fresh weight of shoots and the dry weight of roots, but had no significant effect on the fresh weight of roots and dry weight of shoots. These results indicated that Si treatment could promote the growth of cucumber seedlings to a certain extent even in normal conditions.

**Table 2:** Effect of exogenous Si on the plant height, stem diameter, leaf area, and biomass of cucumber seedlings under cinnamic acid (CA)-induced autotoxicity stress.

Treatment	Plant height (cm)	Stem diameter (mm)	Leaf area (cm^2^)	Shoot		Root	
				Fresh weight (g)	Dry weight (g)	Fresh weight (g)	Dry weight (g)
CK	4.99 ± 0.09 a	4.31 ± 0.08 a	70.23 ± 1.95 a	5.35 ± 0.12 b	0.69 ± 0.01 a	1.12 ± 0.03 a	0.06 ± 0.003 b
Si	5.02 ± 0.10 a	4.34 ± 0.06 a	67.15 ± 2.60 a	5.91 ± 0.11 a	0.71 ± 0.01 a	1.12 ± 0.03 a	0.08 ± 0.002 a
CA	4.43 ± 0.07 c	3.97 ± 0.08 b	36.90 ± 1.59 c	4.30 ± 0.04 d	0.59 ± 0.00 c	0.84 ± 0.02 c	0.04 ± 0.001 d
CA + Si	4.69 ± 0.08 b	4.18 ± 0.06 a	46.38 ± 1.75 b	4.65 ± 0.05 c	0.64 ± 0.02 b	0.99 ± 0.01 b	0.05 ± 0.001 c

Data indicate the mean ± SE (*n* = 3). Different lowercase letters in the same column indicate significant differences according to Duncan’s multiple range test (*p* < 0.05). CK, control.

## Discussion

Plant water status involves regulating the differences between water uptake through roots and leaf transpiration, and maintaining an adequate leaf water status is fundamental to the metabolic activities of plant tissues (Mali and Aery, 2008; Shi et al., 2016). The RWC, water potential, and cell sap concentration of plant leaves are known indicators of their water status. The cell sap concentration of leaves is characterized the consistency of the cell cytoplasm, and a high cell sap concentration is not conducive to the physiological and biochemical processes of cells (Munns et al., 2010). Ferulic acid, a CA derivative, has been reported to significantly inhibit water uptake by seedlings. Reduced water uptake leads to a reduction in leaf water potential and turgor pressure and inhibits seedling growth (Booker et al., 1992). In the present study, CA-induced autotoxicity stress significantly reduced the RWC and water potential of leaves in cucumber seedlings, resulting in an increase in leaf cell sap concentration. These results were consistent with previous findings. Previous studies have demonstrated that exogenous Si can increase leaf water potential and RWC under drought or salt stress in a variety of plants, including rice (Ming et al., 2012; Liu et al., 2019), wheat (Gong and Chen, 2012; Farouk and El-Metwally, 2019), and maize (Amin et al., 2014). In this study, the exogenous addition of Si increased the RWC and leaf water potential and reduced the leaf sap concentration of cucumber leaves. These results indicate that Si helps maintain the normal water status of leaf cells, which is beneficial for the various physiological and biochemical reactions that occur in plant cells. The elevated water potential of Si-treated leaves may be due to the deposition of a double-layered Si cuticle on the leaf epidermis, which reduces water loss from the leaf surface (Ahmed et al., 2014). This may also account for the elevated water potential of Si-treated leaves under CA-induced autotoxicity stress in the present study.

Under autotoxicity stress, plant roots are exposed to and absorb allelochemicals that can disrupt various physiological and biochemical processes, especially water and nutrient uptake (Talukder et al., 2019). Yu and Matsui (1997) reported that the isolates of cucumber root exudates significantly inhibited the uptake of Ca, K, Fe, and Mg by the cucumber root system. Similarly, in the present study, CA-induced autotoxicity stress inhibited the uptake of major (N, P, K, Ca, Mg) and trace elements (Cu, Fe, Mn, Zn) by the leaves and roots of cucumber seedlings to varying degrees. The substances involved in osmotic regulation in plants can be divided into two main categories: organic substances produced by the cells (such as soluble sugars, soluble proteins, and proline) and inorganic ions absorbed from the outside world (such as K, Ca, and Mg; Zhu and Gong, 2014). In addition, mineral elements are of major significance to dry matter accumulation and plant adaptability to adverse conditions. Si has been suggested to play crucial roles in balancing the uptake, transport, and distribution of minerals in plants under drought and salt stress (Rizwan et al., 2015). Numerous studies have indicated that Si treatment promotes the levels of nutrients—such as N (Detmann et al., 2012), P (Zhu and Gong, 2014), K (Farouk and Omar, 2020), Ca (Kaya et al., 2006), Mg (Gunes et al., 2008), Fe (Pavlovic et al., 2013), Zn (Pascual et al., 2016), Mn (Wang and Han, 2007) and Cu (Gunes et al., 2008)—in plant under stress conditions. Our findings are consistent with previous reports. Specifically, we show that under conditions of autotoxicity stress, the levels of major (N, P, K, Ca, Mg) and trace (Fe, Mn, Zn) elements are elevated to varying degrees in the leaves and roots of Si-treated cucumber seedlings. Some researchers have attributed the increased uptake of Ca and to the Si-mediated reduction in plasma membrane permeability and Si-induced increase in plasma membrane H^+^-ATP activity (Liang, 1999; Kaya et al., 2006). These phenomena may also account for the increased Ca and K levels observed in this study. Our results suggest that promoting the uptake and accumulation of mineral elements may be an important nutritional basis for Si application to improve the resistance of cucumber seedlings to autotoxicity stress.

N is a key mineral element involved in plant growth and morphogenesis, and its effective utilization is crucial for plant growth, flowering, fruiting, photosynthesis, and the distribution of photosynthetic products (Prinsi et al., 2009; Nazir et al., 2016). Plants can absorb inorganic N from agroecosystems in various forms, including ammonium nitrogen (NH_4_^+^-N) and nitrate nitrogen (NO_3_^—^N; Carlisle et al., 2012). The most critical enzymes for N metabolism in plants include NR, NiR, GS, GOGAT, and GDH. Following its uptake by plants, NH_4_^+^-N can be directly used for amino acid synthesis. However, NO_3_^−^-N can only be utilized by plants after it has been reduced to NH_4_^+^-N through a reaction catalyzed by NR and NiR (Horchani et al., 2010). NH_4_^+^ is rapidly assimilated into organic N *via* two highly regulated pathways: the GS/GOGAT pathway and the GDH pathway (Zhang et al., 2017). Typically, the GS/GOGAT pathway is the main pathway for NH_4_^+^ assimilation. When the GS/GOGAT pathway is inhibited by stress, the detoxification of NH_4_^+^ by GDH may play an important role (Shao et al., 2015). In addition, stress can severely inhibit N metabolism. Salt stress has been reported to reduce the N concentration of soybean roots and shoots, as well as the activities of GDH, GS, GOGAT, and NR (Farhangi-Abriz and Torabian, 2018). Singh et al. (2015) showed that drought stress reduced N uptake, the activities of GS and GOGAT, and the levels of N-containing compounds in the leaves of sunflower seedling. Consistent with this, our results show that CA-induced autotoxicity stress significantly reduced the activities of NR, NiR, GS, GOGAT, and GDH in cucumber seedlings. The application of exogenous Si can enhance the activity of NiR in pepper plants under drought stress (Pereira et al., 2013). Feng et al. (2010) also reported that Si treatment reduced the limiting effects of heavy metal stress on N metabolism-related enzymes (NR, GS, and GOGAT). In the present study, Si treatment increased the activities of NR, NiR, GS, GOGAT, and GDH in the leaves and roots of cucumber seedlings under autotoxicity stress. However, Si treatment alone had no significant effect on the activities of N metabolism-related enzymes in the leaves and roots of cucumber seedlings under normal conditions. This may be because the beneficial effects of Si on plants were more pronounced under adverse conditions than under normal conditions (Cooke and Leishman, 2016).

Plant roots are not only the first line of defense against autotoxicity, but are also the directly affected by it. Cheng et al. (2016) found that some plant responses to autotoxicity occurred only in the roots, and that the genes relevant to these responses were also expressed only in the roots. However, most studies on autotoxicity stress also focus on leaf photosynthetic parameters, ascorbate-glutathione metabolism, the activities of antioxidant enzymes, and other shoot parameters (Bu et al., 2018; Meng et al., 2021; Zhang et al., 2021). In the present study, CA stress changed the root morphology of cucumber seedlings, resulting in a significant adverse effect on morphological indicators such as total root length, average root diameter, total root volume, total root surface area, and number of branch roots. The inhibitory effects of CA stress on the root system of cucumber seedlings is evident in Figure 5C similar effect of autotoxicity stress has been reported in melons (Zhang et al., 2020b). The Si-induced changes in root morphology observed in this study may be due to increased root elongation as a result of cell wall elongation in the growing region, as reported in sorghum (Hattori et al., 2003). Si is considered a favorable element for plant growth and development, both in normal conditions and under conditions of environmental constraints (Ali et al., 2013). Si can effectively mitigate the effects of heavy metal toxicity in cotton by improving morphological traits such as leaf area, stem length, root length, and the biomasses of roots, shoots, and leaves. It also promotes nutrient uptake by regulating root morphology (Khaliq et al., 2016). In addition, Si has been shown to increase plant height, biomass, RWC, and leaf firmness, and reduce leaf cell death in coriander (Fatemi et al., 2020). Si supplementation not only improves root growth (including morphological traits such as root diameter, root surface area, root volume, total root length, and primary root length), but also the above-ground biomass of plants under abiotic stress (Hameed et al., 2013; Kim et al., 2014). Our findings are consistent with those reported in the above-mentioned studies. That is, the addition of exogenous Si (1 mmol L^−1^) effectively alleviated the inhibitory effects of CA-induced stress on cucumber seedlings by increasing the seedling height, stem thickness, leaf area, and shoot and root biomasses. Based on these findings, we hypothesize that Si-mediated changes in root morphology may be responsible for the increased leaf water status and mineral concentration of cucumber seedlings under CA-induced autotoxicity stress. Maintaining high levels of water status-related parameters in silica-treated plants can be achieved by improving root development to promote water uptake and by forming a silica-cornicle bilayer, a barrier against water loss, on the leaf epidermis (Farouk and Omar, 2020). The higher water status facilitates the enhancement of osmoregulation-maintained metabolic activity, which in turn effectively alleviates the drought and salt stress suffered by the plants (Farouk et al., 2020; Farouk and Omar, 2020). In the present study, in which Si effectively alleviated the adverse effects of CA-induced autotoxicity stress on seedlings, may also be similar to the above study, being that it facilitated the uptake of water and mineral elements through improved root morphology, maintaining a higher leaf water status and ionic homeostasis, and favoring various metabolic activities.

## Conclusion

**Figure 6:**
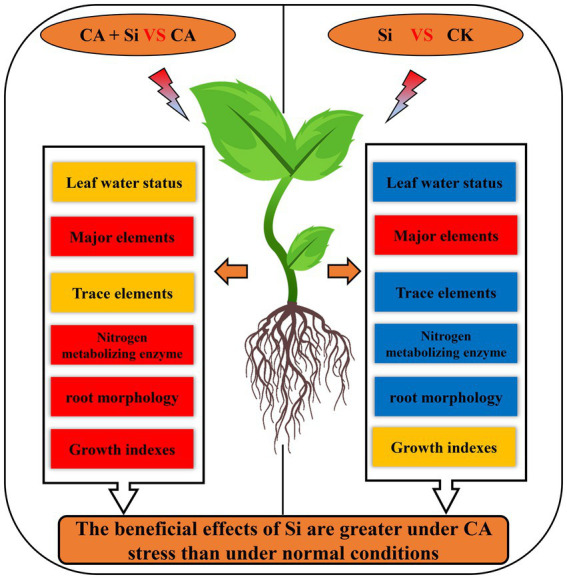
Effects of Si on the shoots and roots of cucumber seedlings under normal conditions and under cinnamic acid-induced autotoxicity stress. Red boxes represent a significant increase in all indicators, orange boxes represent significant increases in some indicators, and blue boxes represent no significant increase in any indicators.

Our study revealed that CA-induced autotoxicity stress was detrimental to the maintenance of normal leaf water status in cucumber seedlings; inhibited the uptake of mineral nutrients and the normal functioning of nitrogen metabolism; and had adverse effects on root morphology, thus suppressing shoot growth. The exogenous application of Si effectively improved the leaf water status, mineral nutrient uptake, activities of N metabolism-related enzymes, and root morphology of the seedlings, thus promoting shoot growth and development. The beneficial effects of Si on the activities of N metabolism-related enzymes in the leaves and roots of cucumber seedlings were greater under CA-induced autotoxicity stress than under normal conditions. As the root system is the first barrier against autotoxicity stress, we speculate that the Si-induced alterations in root morphology are the main reason for improved leaf water status and increased mineral uptake in cucumber seedlings. Our results clearly indicate that Si application at appropriate concentrations is an effective strategy for improving resistance to autotoxicity stress in cucumber seedlings. These findings provide new insights into overcoming CA-induced autotoxicity stress through the widespread use of Si in agricultural production (Figure 6).

## Data availability statement

The original contributions presented in the study are included in the article/supplementary material, further inquiries can be directed to the corresponding author.

## Author contributions

JL, NJ, and JY designed the study. JL, LJ, SW, and XM performed the work. JL was involved with writing the manuscript. JL, NJ, and XM analyzed the data. ZL, XX, ZT, JL, and JY revised the manuscript. All authors contributed to the article and approved the final version of this manuscript.

## Funding

This research was funded by Fuxi Young Talents Fund of Gansu Agricultural University (GAUfx-04Y03); Gansu Top Leading Talent Plan (GSBJLJ-2021-14); Gansu People’s Livelihood Science and Technology Project (20CX9NA099); Industrial support plan project of Gansu Provincial Department of Education (2021CYZC-45); and the Special Project of Central Government Guiding Local Science and Technology Development (ZCYD-2021-07).

## Conflict of interest

The authors declare that the research was conducted in the absence of any commercial or financial relationships that could be construed as a potential conflict of interest.

## Publisher’s note

All claims expressed in this article are solely those of the authors and do not necessarily represent those of their affiliated organizations, or those of the publisher, the editors and the reviewers. Any product that may be evaluated in this article, or claim that may be made by its manufacturer, is not guaranteed or endorsed by the publisher.
